# Knowledge of pressure ulcer prevention: a cross-sectional and comparative study among nurses

**DOI:** 10.1186/1472-6955-6-2

**Published:** 2007-03-09

**Authors:** Mirjam A Hulsenboom, Gerrie JJW Bours, Ruud JG Halfens

**Affiliations:** 1 Maastricht University, Department of Health Care Studies, Section of Nursing Science, The Netherlands; 2 Zuyd University, Center of expertise on Autonomy and Participation, The Netherlands

## Abstract

**Background:**

Pressure ulcers are a common, painful and costly condition. Results of a 1991 study into the knowledge among Dutch hospital nurses on the usefulness of measures to prevent pressure ulcers showed moderate knowledge. Results were confirmed by subsequent studies. In recent years, Dutch guidelines have been updated and the attention given to pressure ulcer care has been increased. This was expected to improve pressure ulcer care and to increase nurses' knowledge. The aims of the current study were to investigate (1) how much nurses employed in Dutch hospitals know about the usefulness of 28 preventive measures considered in the most recent national pressure ulcer guideline; (2) whether differences in knowledge exist between nurses working in hospitals that audit pressure ulcers and those employed in hospitals that do not; and (3) to study whether knowledge among Dutch hospital nurses regarding the usefulness of preventive measures had changed between 1991 and 2003.

**Methods:**

A cross-sectional study design among nurses employed in Dutch hospitals in 2003 was used to investigate their knowledge and differences in knowledge between nurses employed in different types of institution. A comparative design was used to assess whether knowledge differed between this population and that of Dutch hospital nurses in 1991. The nurses' knowledge was assessed by a written questionnaire. Data of 522 respondents meeting the inclusion criteria were analyzed and compared with the results of the 351 nurses included in the 1991 study.

**Results:**

Knowledge in 2003 was slightly better than that in 1991. The nurses were moderately aware of the usefulness of preventive measures. Nurses employed in organizations that monitored pressure ulcers did not display greater knowledge than those employed in organizations that did not do so.

**Conclusion:**

Knowledge among Dutch hospital nurses about the usefulness of measures to prevent pressure ulcers seems to be moderate. Being employed in an institution that monitors pressure ulcer care hardly affects the knowledge level. Knowledge about prevention has improved little since 1991.

## Background

Pressure ulcers are a common problem in health care and represent a significant burden on patients, their relatives and caregivers [1,2]. Annual national prevalence surveys, conducted since 1998, indicate that an average of 18.1% of the patients in hospitals in the Netherlands suffer from pressure ulcers [3,4]. Recent studies conducted in Europe, the United States, Canada and Australia have provided estimates of pressure ulcer prevalence in hospitals ranging from 8.3% to 25.1% [5-9]. The costs of prevention and treatment of pressure ulcers are considerable [10,11]. Estimations for 1998 revealed that more than 1% of the health care budget in the Netherlands was spent on pressure ulcer care. Hence, prevention and proper treatment deserve greater attention [11].

Various preventive measures are being used in nursing practice [1,12]. To address the prevention and treatment of pressure ulcers in a more systematic way, a set of national guidelines based on expert opinions was developed in 1985, and revised in 1992, by the Dutch Institute for Health Care Improvement (CBO) [13,14]. Despite nurses' positive attitudes towards pressure ulcer prevention [15], various studies have revealed a gap between theory and practice [1,16,17].

In 1991, Eggink investigated this gap between theory and practice by assessing nurses' knowledge of the 1985 Dutch national guidelines [18,19]. Although knowledge among health care staff is in itself not enough to ensure implementation of guidelines, it is a prerequisite, along with insight and skills, for the implementation process [20]. The results of the study by Eggink [18] confirmed earlier research revealing that the usefulness of the preventive measures considered in the 1985 national pressure ulcer guideline was insufficiently known among nurses employed in Dutch hospitals. Preventive measures that were not evidence-based were nevertheless still being applied, and sometimes interventions that are known to be useful to prevent pressure ulcers were withheld from patients who needed them. Various other studies have confirmed the lack of knowledge about pressure ulcer prevention. Pieper and Mott [21] showed that registered nurses had insufficient knowledge about pressure ulcers, and Panagiotopoulou and Kerr [22] showed that nurses' average level of agreement with expert opinion regarding the value of preventive measures was a mere 50%. Despite the publication of research findings that support the importance of using evidence-based guidelines, studies have found that guidelines are frequently not implemented and nurses' performance is often based on intuition, experience or habit [23-25].

Because the 1985 and 1992 national guidelines were based on expert opinions rather than evidence, there has been a growing debate about the usefulness of preventive measures. In 2002, a new national set of guidelines was published, based on evidence and expert opinions [12].

Increased consideration is currently being given to pressure ulcers in the Netherlands, as a result of the development of updated pressure ulcer guidelines, publications in professional journals, increased involvement of nurse specialists for pressure ulcers and the implementation of the Annual National Prevalence Pressure Ulcer Surveys [3,4]. In addition, Halfens et al. [26] showed that participation in the Annual Prevalence Surveys resulted in organizations engaging in activities to improve the prevention and treatment of pressure ulcers. Bours et al. [27] described the positive effect of monitoring pressure ulcers each year and giving feedback to hospitals on the use of effective preventive interventions.

These developments might be expected to have resulted in health care institutions and nurses being familiar with the value of preventive measures. In this study, we investigated the development of nurses' knowledge in this respect, to assess whether it has changed over time and whether being employed in an organization that is auditing pressure ulcer prevalence influences nurses' knowledge. The following research questions were formulated:

1. What do nurses employed in Dutch hospitals know about the value of the preventive measures for pressures ulcers considered in the 2002 Dutch Guideline on Pressure Ulcers?

2. Is there a difference in knowledge about the value of the measures to prevent pressure ulcers considered in the 2002 Dutch Guideline on Pressure Ulcers between nurses employed in hospitals that participate in the Annual National Prevalence Surveys and nurses employed in non-participating institutions?

3. Is there a difference between the knowledge among nurses employed in Dutch hospitals in 1991 and those employed in 2003 as regards the value of the preventive measures for pressure ulcers considered in the Dutch Guideline on Pressure Ulcers?

## Methods

### Design

A cross-sectional design was used to assess nurses' knowledge in 2003. The subjects were nurses employed in Dutch hospitals. The difference between nurses' knowledge about preventive measures in 2003 and 1991 was assessed by combining the database of the 2003 study with that of a comparable study conducted in 1991 [18,19].

### Population sample and procedure

The 2003 survey population consisted of 2 samples. Sample 1 (referred to as Non-NPS) was derived from the mailing list of the 976 subscribers to the Dutch professional journal *Verpleegkunde Nieuws *('Nursing News'), who were receiving the journal at their home address. This mailing list covered 10% of the nurses in the Netherlands. Nurses were approached by letter, asking them to fill in a questionnaire. Sample 2 (referred to as NPS) was obtained by randomly contacting 23 of the 48 hospitals that participated in the 2003 National Prevalence Survey (NPS). Contact persons of these hospitals were requested to each approach five nurses from five departments in their institutions and ask them to complete the questionnaire. This sample was recruited two months after the 2003 NPS. In view of the impact of the NPS (in terms of e.g. preparations, actual measurements and involvement of nurses) on participating departments and staff, it was expected that the nurses from the institutions participating in the NPS would have greater knowledge about pressure ulcer prevention and therefore differ from the nurses in sample 1.

Nurses from both samples received an accompanying letter with information about the study and an explanation of the method.

The 1991 study [18,19] also used a random sample from the subscribers to *Verpleegkunde Nieuws *(which was at that time distributed to nurses free of charge) who were employed by hospitals. At that time, the journal's mailing list covered 80% of the nurses in the Netherlands. Nurses were approached by mail to complete the questionnaire.

Both in 1991 and in 2003, the inclusion criteria for participation were that respondents (1) had to be qualified nurses; (2) had to be employed by a university hospital or general hospital in the Netherlands; (3) had to be directly involved in patient care; (4) had to have answered more than 10% of the questions regarding the preventive measures. Since two samples were drawn in 2003, it was possible that some nurses received the questionnaire twice. To avoid misclassification, respondents were excluded if they answered the question 'Have you received and returned this questionnaire before?' affirmatively.

### Questionnaires

The 2003 and 1991 studies used comparable written questionnaires to collect data. The 2003 Pressure Ulcer Questionnaire (PUQ-2003) measured the use of and beliefs and knowledge about preventive measures among health care staff employed in various settings. The present study analyzed the demographic questions and the knowledge of nurses employed in hospitals.

The 2003 questionnaire was based on the questionnaire used in the 1991 study and adapted to include the measures mentioned in the 2002 national Guideline on Pressure Ulcers [12]. This guideline classified preventive measures into two categories. The first category includes 15 measures that are useful to prevent pressure ulcers for all patients at risk, such as repositioning patients every 3 hours. The second category comprises 13 measures that are not useful to prevent pressure ulcers, such as using a sheepskin. Useful and non-useful measures are labelled as recommended and non-recommended interventions for preventing pressure ulcers, respectively. All of these measures, plus the measures that were included in the 1991 questionnaire but not in the 2002 guideline, were included in the 2003 questionnaire (see table 2). In all, the questionnaire asked nurses to evaluate 28 measures (15 useful and 13 not useful) in terms of their being 'useful', 'sometimes useful', 'not useful' or 'don't know'.

**Table 2:** Classification of preventive measures: knowledge about usefulness in 2003 and 1991

	**2003 (n = 522)**				**1991 (n = 351)**				
	
**USEFUL PREVENTIVE MEASURES**	Useful	Some-times	Not useful	Don't know	Useful	Some-times	Not useful	Don't know	**p-value**
Preventing maceration of the skin	**90.4**	9.0	0.0	0.6	**89.1**	9.7	0.3	0.9	0.58
Ensuring a clean, dry and square lower layer of bedclothes	**98.5**	1.5	0.0	0.0	**99.7**	0.3	0.0	0.0	0.05
Helping non-bedridden patients lift up or assume a different position	**73.6**	23.1	1.2	2.1	**62.1**	29.6	3.7	4.6	0.00*
Involving patients in prevention	**79.7**	20.3	0.0	0.0	**58.0**	40.3	0.6	1.1	0.00*
Assessing risk by means of an instrument and clinical judgment	**92.5**	6.7	0.2	0.6	**36.9**	35.1	7.7	20.3	0.00*
Using air mattresses and pillows	**68.1**	28.6	1.2	2.1	43.6	**49.9**	3.7	2.8	0.00*
Using viscoelastic (foam) mattresses and pillows	**35.0**	33.1	3.5	28.3	21.4	**48.0**	13.1	17.4	0.00*
Smearing the skin with topical agents to prevent dehydration	**36.1**	43.6	13.7	6.6	60.0	**33.4**	3.4	3.1	0.44
Involving family/friends/caregivers in prevention	**65.1**	34.0	0.4	0.6	26.2	**63.5**	5.4	4.8	0.75
Assessing nutritional state and preventing nutritional deficiency	**94.3**	5.6	0.0	0.2	**86.9**	12.5	0.3	0.3	0.00*
Ensuring good hygiene	**98.1**	1.3	0.6	0.0	**99.7**	0.3	0.0	0.0	0.06
Using a 30-degree side to side turn at least every 4 hours	**48.9**	42.0	7.1	1.9	-	-	-	-	-
Avoiding contact of the heels with the lower layer by putting a pillow under the lower legs	**40.0**	44.1	12.6	3.3	-	-	-	-	-
Preventing shear forces	**95.6**	4.4	0.0	0.0	-	-	-	-	-
Smearing the skin with topical agents in case of urine and/or faeces incontinence	**52.0**	37.4	6.7	3.8	-	-	-	-	-

**Mean number of measures judged correctly**	**10.6 of 15 (70.6%)**				**7.2 of 11 (65.4%)**				0.00*
**σ and range**	**2.11; 2–15**				**1.49; 3–11**				
**% having sufficient knowledge (judged ≥ 70% of measures correct)**	**56.6%**				**43.0%**				0.00*

**NON-USEFUL PREVENTIVE MEASURES**	Useful	Some-times	Not useful	Don't know	Useful	Some-times	Not useful	Don't know	**p-value**

Using a cradle	14.1	71.5	**10.9**	3.5	4.8	**79.5**	8.8	6.8	0.00*
Reactivation and mobilisation by paramedics	76.8	22.8	**0.2**	0.2	52.7	**45.9**	0.6	0.9	0.00*
Wrapping the heels/elbows in natural cotton wool and bandages	4.6	29.6	**54.1**	11.7	14.8	68.7	**14.0**	2.6	0.00*
Using ice compresses	0.2	5.1	**76.5**	18.2	1.4	32.2	**52.4**	14.0	0.00*
Using warm compresses	0.2	6.2	**85.4**	8.3	4.0	40.2	**44.4**	11.4	0.00*
Massage	18.3	40.0	**35.6**	6.2	70.5	25.5	**2.0**	2.0	0.00*
Using water mattresses and pillows	23.3	41.4	**16.4**	18.9	39.6	**48.7**	4.6	7.1	0.00*
Using gel mattresses and pillows	39.2	45.5	**5.2**	10.2	20.6	**57.3**	4.9	17.2	0.00*
Inserting a catheter to prevent maceration of the skin	29.2	62.3	**5.0**	3.5	14.0	79.8	**4.6**	1.7	0.77
Smearing the skin (with topical agents) to prevent disturbance in blood supply caused by pressure	19.6	34.2	**35.0**	11.2	69.6	21.5	**3.7**	5.2	0.00*
Using a 90-degree side to side turn at least every 4 hours	68.1	29.4	**0.2**	2.3	-	-	-	-	-
Avoiding contact of heels with lower layer by using ring-shaped cushions or gloves filled with water	10.4	38.2	**42.0**	9.4	-	-	-	-	-
Using a sheepskin	2.7	30.1	**62.8**	4.4	-	-	-	-	-

**Mean number of measures judged correctly**	**4.2 of 13 (32.3%)**				**3.5 of 10 (35.0%)**				0.00*
**σ and range**	**2.23; 0–10**				**1.60; 0–8**				
**% having sufficient knowledge (judged ≥ 70% of measures correct)**	**0.2%**				**3.1%**				0.00*

Because of the small number, missing values are not presented in the table

Correct answers are shown in bold print

* difference is significant (2-tailed)

Although the 1991 questionnaire (PUQ-1991) was comparable, this questionnaire was based on the 1985 Consensus on Pressure Sore Prevention. In contrast to the 2002 guideline, the 1985 version distinguished three categories: useful (9 preventive measures), sometimes useful (11 measures) and non-useful (7 measures). After some demographic questions, the 1991 questionnaire asked nurses to evaluate these 27 measures in terms of their being 'useful', 'sometimes useful', 'not useful' or 'don't know'.

### Analysis

Descriptive statistics were used to assess the nurses' knowledge about the usefulness of preventive measures in 2003.

To compare the knowledge between groups (nurses employed in institutions participating in the National Prevalence Survey (NPS) versus nurses employed in non-participating institutions (Non-NPS) and 1991 versus 2003), answers were recoded as 'judged correctly' or 'judged incorrectly'. Nurses were said to have judged a measure correctly if their judgement corresponded with the recommendations in the guidelines that were in force at the time. Differences in knowledge about the usefulness of each individual measure were examined by Chi-squared tests. Fisher exact tests were used if expected cell counts were less than 5.

The differences in knowledge about all measures of each category (useful and not useful) together were examined by means of t-tests for the mean scores for each category. In view of the difference in the number of measures tested in 2003 and 1991, overall scores were made comparable on a 15-point and a 13-point scale respectively, to test whether the overall knowledge about these measures differed between the groups.

Having sufficient knowledge was defined as correctly judging 70% or more of the measures in each category (useful and not useful). Two-sided Chi-square tests were used to compare the two groups as regards the percentage of nurses having sufficient knowledge.

Logistic regression analyses for the categories 'useful' and 'not useful' were used to examine which factors determined any significant differences found between the 2003 and 1991 groups as regards having sufficient knowledge. The analysis included age, work experience, educational level, work setting and 'being a respondent in 1991 or 2003'. For specification of the demographic variables, see table 1.

**Table 1:** sample characteristics

	**2003 (n = 522)**	**1991 (n = 351)**	**Significance**
	
	**n (%)**		**p-value**
**Gender**			
Male	77 (14.8)	41 (11.7)	0.07
Female	437 (83.7)	309 (88.0)	
**Educational level**			
Enrolled nurse	375 (71.8)	328 (93.4)	0.00
Registered nurse	147 (28.2)	23 (6.6)	
**Work setting**			
General hospital	449 (86.0)	279 (79.5)	0.01
University hospital	73 (14.0)	72 (20.5)	
**Work experience**			
< 5 yrs	176 (33.8%)	124 (35.6%)	0.00
5–15 yrs	164 (31.5%)	174 (50.0%)	
> 15 yrs	181 (34.7%)	50 (14.4%)	
**Age**			
20–25 yrs	111 (21.3%)	55 (15.7%)	0.00
25–30 yrs	114 (21.8%)	124 (35.3%)	
30–35 yrs	67 (12.8%)	73 (20.8%)	
35–40 yrs	63 (12.1%)	37 (10.5%)	
40–45 yrs	69 (13.2%)	38 (10.8%)	
45–50 yrs	45 (8.6%)	12 (3.4%)	
50–55 yrs	42 (8.0%)	7 (2.0%)	
> 55 yrs	11 (2.1%)	5 (1.4%)	

In order to investigate whether the classification of the nurses' answers to PUQ-2003 corresponded to the classification that was expected in terms of the categories of usefulness used in the 2002 national guideline (i.e., useful and not useful), Principal Factors Analyses (PFA) with Varimax rotation was applied to the dichotomized variables. The Cattell scree test was used to examine the number of factors that reflect the underlying structure of the questionnaire. Subsequently, the percentage of explained variance of the rotated model was calculated for the solution derived from the Cattell scree test and the expected solution. If items did not load (i.e. < 0.2) on any factor, we concluded that the usefulness of these measures was not or only moderately known among the research population.

Data were analysed using SPSS 12.01 for Windows. In view of the risk of type I errors, differences in all analyses were considered significant at alpha ≤ 0.01.

## Results

### Response rate

The 2003 questionnaire was sent to 1626 nurses. This sample consisted of 976 nurses, employed in various settings, who had been selected on the basis of their subscription to the Dutch professional nursing journal *Verpleegkunde Nieuws *(this sample being referred to below as Non-NPS) and 750 nurses selected because they were employed by hospitals that participated in the 2003 Annual National Prevalence Survey (referred to as NPS) [3]. After 12 weeks, 729 questionnaires had been returned (44.8%), 97 of which had not (24) or only partially (55) been completed. In the end, 650 questionnaires remained (39.9%), 522 of which (32.1%) were used for the analyses because they met the inclusion criteria (14.5% of the Non-NPS sample and 50.7% of the NPS sample). The main reason for excluding questionnaires were that respondents were not involved in patient care, were not working in a hospital or had completed less than 90% of the questions on knowledge about preventive measures. None of the nurses who completed and returned the questionnaire reported that they had received and returned this questionnaire before.

The 1991 study population consisted of 730 nurses, selected on the basis of their subscription to *Verpleegkunde Nieuws *and their being employed by a Dutch hospital. Of the 556 (76.1%) questionnaires that were returned, 205 respondents did not meet the inclusion criteria or did not fully complete the questionnaire. In the end, 351 (48.0%) questionnaires were used for further analyses.

### Sample characteristics

A sample of 522 nurses met the inclusion criteria of the 2003 study, while 351 nurses had met those of the 1991 study. Compared to 1991, the 2003 sample of nurses differed in all characteristics, except gender (see table 1). Compared to 1991, the 2003 sample included older nurses and more registered nurses, as well as more nurses who were employed in a general hospital. Nurses in 2003 were also more experienced. The characteristics of the NPS and non-NPS samples only differed significantly as regards work experience (p = 0.001), with more experienced nurses in the NPS group.

### Knowledge in 2003

Table 2 presents the results regarding nurses' knowledge about preventive measures for the 2003 sample. It shows that most nurses knew that these measures are recommended as useful for patients at risk of pressure ulcer development: an average of 10.6 of the 15 useful measures mentioned in the 2002 Guideline on Pressure Ulcers were marked as useful. Compared to the useful measures, the nurses in the 2003 sample were less aware of the non-useful preventive measures: nurses judged an average of 4.2 of these 13 measures as not useful. A considerable percentage of the nurses regarded various non-useful preventive measures as sometimes useful. And although the guidelines recommend not using '90-degree side to side turn' and 'reactivation and mobilisation by paramedics', the majority of the nurses evaluated these as useful.

#### Comparison between the NPS and Non-NPS groups

Chi-square tests indicated that the knowledge about the usefulness of individual measures was comparable between the NPS and Non-NPS groups (see table 3). Only the differences in the correct judgement of 'using ice compresses' and 'using warm compresses' were nearly significant, with nurses in the NPS group more likely to judge these measures correctly (p = 0.034 and p = 0.039, respectively).

**Table 3:** Knowledge of preventive measures for nurses employed in hospitals participating in the National Prevalence Measurements (NPS) and nurses employed in non-participating organizations (non-NPS) (%)

	**NPS (n = 380)**				**Non NPM (n = 142)**				
	
**USEFUL PREVENTIVE MEASURES**	Useful	Some-times	Not useful	Don't know	Useful	Some-times	Not useful	Don't know	**p-value**
Prevent maceration of the skin	**92.1**	7.4	0.0	0.5	**85.9**	13.4	0.0	0.7	0.05
Take care for a clean, dry and square lower layer of bedclothes	**98.4**	1.6	0.0	0.0	**98.6**	1.4	0.0	0.0	0.73
Helping non-bedridden patients lift up or assume a different position	**73.1**	23.2	0.8	2.9	**75.0**	22.9	2.1	0.0	0.81
Involving the patient in prevention	**78.4**	21.6	0.0	0.0	**83.1**	16.9	0.0	0.0	0.23
Assessing risk by means of an instrument and clinical judgment	**93.1**	6.1	0.3	0.5	**90.8**	8.5	0.0	0.7	0.50
Using air mattresses and pillows	**68.9**	28.2	1.1	1.8	**66.2**	29.6	1.4	2.8	0.58
Using visco elastic (foam) mattresses and pillows	**36.1**	33.2	3.8	26.9	**32.1**	32.9	2.9	32.1	0.47
Smearing the skin with topical agents to prevent dehydration	**37.8**	42.8	14.4	5.1	**31.7**	45.8	12.0	10.6	0.22
Involving family/friends/caregivers in prevention	**63.9**	35.4	0.3	0.5	**68.3**	30.2	0.7	0.7	0.49
Assessing nutritional state and preventing a nutritional deficiency	**94.2**	5.8	0.0	0.0	**94.4**	4.9	0.0	0.7	0.94
Ensuring good hygiene	**97.9**	1.6	0.5	0.0	**98.6**	0.7	0.7	0.0	0.49
Using a 30-degree side to side turn at least every 4 hours	**48.8**	43.3	7.1	0.8	**49.3**	38.6	7.1	5.0	0.98
Avoid contact of the heels with the lower layer by putting a pillow under the lower legs	**42.2**	43.2	11.8	2.6	**33.8**	46.5	14.8	4.9	0.07
Preventing shearing forces	**95.0**	5.0	0.0	0.0	**97.2**	2.8	0.0	0.0	0.23
Smearing the skin with topical agents in case of urine and/or faeces incontinence	**52.2**	38.3	6.6	2.9	**51.4**	35.2	7.0	6.3	0.88

**Mean number of measured judged correctly**	**10.6 (70.6%)**				**10.5 (70.0%)**				0.46
**σ and range**	**2.19; 2–15**				**1.88; 3–15**				
**% having sufficient knowledge (judged ≥ 70% of measures correct)**	**58.4%**				**51.4%**				0.15

**NOT USEFUL PREVENTIVE MEASURES**	Useful	Some-times	Not useful	Don't know	Useful	Some-times	Not useful	Don't know	**p-value**

Using a cradle	13.4	72.6	**11.3**	2.7	15.7	68.6	**10.0**	5.7	0.69
Reactivation and mobilisation by paramedics	74.9	24.5	**0.3**	0.3	81.7	18.3	**0.0**	0.0	0.54
Wrapping the heels/elbows in natural cotton wool and bandages	4.5	30.1	**54.9**	10.6	4.9	28.2	**52.1**	14.8	0.59
Using ice compresses	0.3	4.6	**79.0**	16.1	0.0	6.5	**69.8**	23.7	0.03
Using warm compresses	0.0	5.0	**87.5**	7.4	0.7	9.2	**79.6**	10.6	0.03
Massage	19.2	41.3	**33.7**	5.8	15.7	36.4	**40.7**	7.1	0.17
Using water mattresses and pillows	22.2	41.8	**18.3**	17.7	26.2	40.4	**11.3**	22.0	0.05
Using gel mattresses and pillows	39.3	45.6	**6.1**	9.0	38.7	45.1	**2.8**	13.4	0.13
Inserting a catheter to prevent maceration of the skin	30.5	62.6	**4.8**	2.1	25.7	61.4	**5.7**	7.1	0.67
Smearing the skin (with topical agents) to prevent disturbance in blood supply caused by pressure	21.4	33.6	**35.4**	9.5	14.8	35.9	**33.8**	15.5	0.75
Using a 90-degree side to side turn at least every 4 hours	67.6	30.8	**0.3**	1.3	69.3	25.7	**0.0**	5.0	0.54
Avoid contact of heels with lower layer by using ring-shaped cushions or gloves filled with water	10.8	35.4	**44.3**	9.5	9.2	45.8	**35.9**	9.2	0.08
Using a sheepskin	2.9	28.2	**64.1**	4.7	2.1	35.2	**59.2**	3.5	0.31

**Mean number of measured judged correctly**	**4.3 (33.0%)**				**3.9 (30.0%)**				0.10
**σ and range**	**2.14; 0–10**				**2.44; 0–9**				
**% having sufficient knowledge (judged ≥ 70% of measures correct)**	**0.3%**				**0.0%**				0.54

Because of the small number, missing values are not presented in the table

Correct answers are highlighted in bold

* difference is significant (2-tailed)

T-tests for the mean number of correctly judged measures indicated that the overall score was comparable between the two groups (10.6 versus 10.5 useful and 4.3 versus 3.9 not useful). Comparable results were obtained when comparing the two groups in terms of the percentage of nurses having sufficient knowledge (i.e. judging ≥ 70.0% of the measures in each category correctly; p = 0.15 for the useful measures category and p = 0.54 for the non-useful measures category).

### Comparison of knowledge in 2003 and 1991

Chi-square tests for dichotomized measures ('judged correctly' and 'judged incorrectly') indicated that in 2003, nurses were more likely to evaluate the measures in line with the guideline in force at the time than in 1991 (see table 2). For instance, in 1991, 36.9% of the nurses judged the 'risk assessment' measure in accordance with the 1985 national guideline, whereas in 2003, 92.5% judged this measure correctly according to the 2002 guideline. However, not all measures for which there were significant differences were evaluated more correctly in 2003 than in 1991. For instance, the usefulness of various types of mattress and pillow (except air mattresses) was better known among the 1991 sample than the 2003 sample. Besides, the comparable distributions of answers in 1991 and 2003 for 'using a cradle' and 'reactivation and mobilisation by paramedics' indicate that nurses' knowledge of these measures has not been adapted to the altered guidelines. Despite the significant general increase in knowledge about five non-useful preventive measures, a large number of nurses still judged these measures incorrectly in 2003. For example 'smearing the skin to prevent disturbance in blood supply' was evaluated correctly by 35.0% in 2003, against 3.7% in 1991. However, 53.8% of the nurses in 2003 still said that it is sometimes or always a useful measure.

T-tests showed that, on average, the nurses surveyed in 2003 correctly judged significantly more useful measures than the nurses in the 1991 study (p = 0.00). In 1991, however, nurses correctly judged more non-useful measures (p = 0.00).

Similar results were found when comparing the percentages of nurses having sufficient knowledge. Chi-square tests indicated that the differences for the categories 'useful' (56.5% in 2003 versus 43.0% in 1991) and 'not useful' (0.2% in 2003 versus 3.1% in 1991) were both significant (p = 0.00).

Since the 2003 and 1991 samples differed in their characteristics, multivariate logistic regression analyses were conducted to find factors that could explain the significant differences in the percentage of nurses having sufficient knowledge (in the sense of having judged ≥ 70% of the measures correctly), for each category of variables. Results show that the differences in these two categories do not seem to have been influenced by demographic characteristics, only by belonging to a particular group. Nurses involved in the 2003 survey were 67.0% more likely to have sufficient knowledge about the category of useful measures than the nurses in the 1991 study (Exp(B) = 1.670l; p < 0.00; 95% C.I. = 1.269–2.197). Apparently, their level of up-to-date knowledge was higher than that of the nurses in the 1991 study. Regarding the category of non-useful measures, the opposite was found: nurses in 1991 were better informed about the value of these measures (Exp(B) = 0.059, p = 0.007; 95% C.I. = 0.008–0.462).

### Confirmatory analysis

The recommendations in the 2002 Guideline on Pressure Ulcers are divided into useful and non-useful preventive measures. To assess whether the nurses' knowledge in 2003 made the same distinction, we used Principal Factor Analysis with a two-factor solution. The solution explained 13.1% of the variance. A scree plot and an analysis with a three-factor solution confirmed the adequacy of this solution. Although a solution with three factors explained more variance of the model (Eigenvalues: 2.133; 1.521; 0.889), the extra factor only explained a small part (see table 4). Therefore, we opted for the two-factor solution. Table 4 shows that nurses made a distinction comparable to that used in the guideline, with factor 1 apparently closely associated with the non-useful preventive measures and factor 2 with the useful measures. Five measures did not load on any factor. This is confirmed by the results in table 2. 'Using viscoelastic mattresses' and 'smearing the skin to prevent dehydration' were judged correctly by 35.0% and 36.1% of the nurses, respectively, and the majority of the nurses evaluated 'using a cradle' as sometimes useful. And 'using a 90-degree side to side turn' and 'reactivation and mobilisation by paramedics' were considered useful to prevent pressure ulcers. Apparently, there were differences of opinion between nurses about the value of these five measures.

**Table 4:** Factor Matrix (factorloadings > 0.2)

	**Factor 1**	**Factor 2**
Prevent maceration of the skin		0.370
Take care for a clean. dry and square lower layer of bedclothes		0.343
Helping non-bedridden patients lift up or assume a different position		0.531
Involving the patient in prevention		0.219
Assessing risk by means of an instrument and clinical judgment		0.274
Using air mattresses and pillows		0.242
Using visco elastic (foam) mattresses and pillows		
Smearing the skin with topical agents to prevent dehydration		
Involving family/friends/caregivers in prevention		0.409
Assessing nutritional state and preventing a nutritrional deficiency		0.456
Ensuring good hygiene		0.285
Using a 30-degree side to side turn at least every 4 hours		0.222
Avoid contact of the heels with the lower layer by putting a pillow under the lower legs		0.200
Prevent shear forces		0.392
Smearing the skin with topical agents in case of urine and/or faeces incontinence		0.218
Using a cradle		
Reactivation and mobilisation by paramedics		
Wrapping the heels/elbows in natural cotton wool and bandages	0.578	
Using ice compresses	0.494	
Using warm compresses	0.495	
Massage	0.422	
Using water mattresses and pillows	0.316	
Using gel mattresses and pillows	0.265	
Inserting a catheter to prevent maceration of the skin	0.204	
Smearing the skin (with topical agents) to prevent disturbance in blood supply caused by pressure	0.463	
Using a 90-degree side to side turn at least every 4 hours		
Avoid contact of the heels with the lower layer by using ring-shaped cushions or gloves filled with water	0.577	
Using a sheepskin	0.464	
	
Eigenvalue (% of variance)	2.137 (7.6%)	1.546 (5.5%)

## Discussion

The present study used a cross-sectional design to examine current knowledge among nurses regarding the usefulness of measures to prevent pressure ulcers. In addition, a comparative design was used to assess whether nurses' knowledge had changed between 1991 and 2003. Secondary analyses were conducted to test whether nurses' knowledge about the usefulness of preventive measures used the same classification as that used in the national guideline.

The results of the present study show that the knowledge among nurses employed in Dutch hospitals about the usefulness of preventive measures mentioned in the national Guideline on Pressure Ulcers [12] is moderate. Knowledge about non-useful measures in particular seems to be poorly disseminated, whereas useful measures are better known among nurses. In addition, we found that being employed in a hospital that monitors pressure ulcers annually does not lead to better knowledge about preventive measures.

Comparing our results with a similar study conducted in 1991 [18,19], we can conclude that despite the increased attention and new developments in the area of pressure ulcer care, knowledge about useful measures has not greatly increased. A considerable number of nurses still evaluated useful measures incorrectly in 2003, and the knowledge about non-useful measures had actually decreased. Against our expectations, results showed that the usefulness of a few measures was better known in 1991 than in 2003. Apparently, nurses in 2003 were not aware of the changes in the guidelines regarding the usefulness of these measures, which in the 1985 Consensus on Pressure Sore Prevention were regarded as 'sometimes useful', while in the 2002 version they are regarded as 'not useful'. Nurses' judgements on these measures in 2003 were comparable to those of the nurses in 1991. It seems likely that the nurses in the 2003 sample had not familiarized themselves with the updated guidelines or had not received up-to-date information about pressure ulcer prevention.

We used a 70% cut-off point (i.e. judging 70% of the measures correctly) to identify nurses having sufficient knowledge, which could be regarded as mild. Even at this cut-off level, however, only a small number of nurses met the criterion. A higher cut-off point, making the criterion stricter, would have led to even more nurses being labelled as not having sufficient knowledge. Nevertheless, the results obtained with the current cut-off point give some indication of the level of knowledge.

Apparently, the efforts to disseminate knowledge about the prevention of pressure ulcers have not led to major improvements in knowledge. Although it might be thought that the difference in the percentage of nurses with sufficient knowledge could be explained by demographic differences between the 2003 and 1991 study populations, logistic regression analyses showed that none of the demographic variables had a significant influence.

Our figures may even overestimate the nurses' actual knowledge. It is known that postal surveys are susceptible to positive selection bias [28,29], and respondents tend to differ from the underlying population [30]. In view of the response rate and composition of the sample in the 2003 study and the difference in availability and coverage of the *Verpleegkunde Nieuws *professional journal between 1991 and 2003, it seems likely that it was especially the more interested nurses who responded in the 2003 study, which may have resulted in a biased impression of the knowledge of nurses in general. The average actual knowledge among all nurses is therefore probably poorer. Nevertheless, the results give some idea of the knowledge among nurses employed in hospitals.

To avoid misclassification of the two 2003 samples (NPS and Non-NPS), the questionnaire also asked 'Have you received and returned this questionnaire before?'. However, because of the method used to recruit nurses for the NPS sample (asking contact persons of a random selection of NPS hospitals to each approach five nurses from five departments in their institutions), it is possible that nurses from the Non-NPS sample (recruited through their subscriptions to a professional journal) were also working in an NPS hospital but were not selected by the contact person. These nurses are expected to be better informed about pressure ulcer care than Non-NPS nurses who are not working in an NPS hospital. This means that the results of the Non-NPS sample may be biased, in that these nurses' knowledge may have been overestimated. However, in view of the small coverage of the *Verpleegkunde Nieuws *journal in 2003, the authors think that the chance that these results are biased, though real, is probably small.

Results of the present study confirm those of Pieper and Mott [21] and Panagiotopoulou and Kerr [22]. Sinclair et al. [31] and Gunningberg [32], who assessed the knowledge among nurses before implementing an educational programme, also reported that knowledge regarding pressure ulcer prevention among nurses was moderate.

With hindsight, use of the term 'viscoelastic mattresses and pillows' may have caused information bias. A considerable proportion of the nurses answered 'don't know' to this question, or did not answer the question at all. A more detailed description or brand names of these support surfaces would probably have changed the distribution of answers and provided a better idea of the knowledge about these devices.

Principal Factors Analyses showed that a factor solution consistent with the two-category classification of preventive measures in the 2002 national guideline only explained 13.1% of the variance of the construct we measured. Streiner and Norman [33] provide an explanation for these outcomes. Whereas in most situations, measured variables are defined by the construct and are expected to be correlated, in our study it was the variables which defined the construct: the knowledge about usefulness was defined by the individual questions about preventive measures. Despite the low degree of explained variance, the specific variables do matter. Our Principal Factors Analyses therefore have to be regarded as confirmatory, in that they were intended to test whether nurses' knowledge had the same classification as the classification used in the national guideline. Another explanation for our findings could be the poor agreement in judgments about the usefulness of preventive measures. A result showing more convergent validity would probably have been obtained if nurses' opinions about usefulness had been more homogeneous.

Apparently, the preventive measures recommended in the guidelines are not very well known among nurses. Pressure ulcer prevention guidelines rarely seem to be based on scientific evidence, but rather on expert opinion. Moreover, the dissemination of knowledge among nurses is also influenced by the known barriers to the use of guidelines, like lack of staff and time [15], and probably by the quality of the guidelines. For instance, measures such as 'using a cradle' and 'reactivation and mobilisation by paramedics' are not clearly classified as non-useful by the 2002 Dutch Guideline on Pressure Ulcers. Although experts consider them not useful, they acknowledge that they may be useful in some cases. The equivocal expert judgments regarding massage [34] and the use of sheepskins [35] cause confusion as well. Therefore, the distribution of answers (tables 2 and 3) is not surprising and the conclusions about correct judgments have to be interpreted with caution. For this reason, we recommend further research to test the effectiveness of these preventive measures and to implement more evidence-based guidelines instead of opinion-based guidelines.

The implementation of guidelines and their translation into practice remains difficult [36]. Grol and Wensing [37] stated that the process of implementing guidelines is influenced by several factors, including interest and commitment to guideline development. In 2005, Clarke et al. [38] studied which strategy would be best to effectively implement pressure ulcer guidelines. They concluded that implementing these guidelines demands a comprehensive approach. To increase nurses' knowledge about prevention of pressure ulcers, guidelines should be implemented in a systematic and meticulous way. Constant attention to prevention remains essential. In the Netherlands, the government has taken an important step by considering the presence of pressure ulcers as a quality indicator of care. With adequate policy and management, awareness among health care organizations of the need to reduce the prevalence of pressure ulcers will hopefully affect the level of awareness among their staff. However, based on the present study and the research by Buss et al. [23], which found that nurses do not seem to have the intention to change their preventive actions, we recommend further research to study which tailored approach would help change nurses' knowledge, beliefs and performance regarding pressure ulcer prevention. Potentially useful interventions in this respect include education and refresher courses for nurses in the context of a comprehensive approach.

## Conclusion

In conclusion, Dutch hospital nurses' knowledge regarding the usefulness of measures to prevent pressure ulcers seems to be moderate. Being employed in an organization that monitors pressure ulcer care hardly affects this knowledge. Besides, knowledge about prevention has not improved over time. Apparently, activities undertaken to reduce the prevalence of pressure ulcers have had little or no effect on nurses' knowledge. Adequate dissemination of pressure ulcer prevention guidelines seems a prerequisite to improving the quality of pressure ulcer prevention.

## Competing interests

The author(s) declare that they have no competing interests.

## Authors' contributions

MH carried out the study, performed the statistical analysis and drafted the manuscript. GB and RH participated in the design of the study and drafted the manuscript. All authors read and approved the final manuscript.

## Pre-publication history

The pre-publication history for this paper can be accessed here:


